# Prognostic Mutational Signatures of NSCLC Patients treated with chemotherapy, immunotherapy and chemoimmunotherapy

**DOI:** 10.1038/s41698-023-00373-0

**Published:** 2023-03-27

**Authors:** Margaret R. Smith, Yuezhu Wang, Ralph D’Agostino, Yin Liu, Jimmy Ruiz, Thomas Lycan, George Oliver, Lance D. Miller, Umit Topaloglu, Jireh Pinkney, Mohammed N. Abdulhaleem, Michael D. Chan, Michael Farris, Jing Su, Kathryn F. Mileham, Fei Xing

**Affiliations:** 1 Department of Cancer Biology, Wake Forest University School of Medicine, Winston-Salem, NC 27157 USA; 2 Department of Biostatistics and Data Science, Wake Forest University School of Medicine, Winston-Salem, NC USA; 3 Department of Hematology and Oncology, Wake Forest University School of Medicine, Winston-Salem, NC USA; 4 Department of Pharmacy, Atrium Health Wake Forest Baptist Medical Center, Winston-Salem, NC USA; 5 Department of Biology, Winston Salem State University, Winston-Salem, NC USA; 6 Department of Radiation Oncology, Wake Forest University School of Medicine, Winston-Salem, NC USA; 7 Department of Biostatistics, Indiana University School of Medicine, Indianapolis, IN USA; 8 Department of Solid Tumor Oncology, Levine Cancer Institute, Atrium Health, Charlotte, NC USA

**Keywords:** Prognostic markers, Cancer genetics

## Abstract

Different types of therapy are currently being used to treat non-small cell lung cancer (NSCLC) depending on the stage of tumor and the presence of potentially druggable mutations. However, few biomarkers are available to guide clinicians in selecting the most effective therapy for all patients with various genetic backgrounds. To examine whether patients’ mutation profiles are associated with the response to a specific treatment, we collected comprehensive clinical characteristics and sequencing data from 524 patients with stage III and IV NSCLC treated at Atrium Health Wake Forest Baptist. Overall survival based Cox-proportional hazard regression models were applied to identify mutations that were “beneficial” (HR < 1) or “detrimental” (HR > 1) for patients treated with chemotherapy (chemo), immune checkpoint inhibitor (ICI) and chemo+ICI combination therapy (Chemo+ICI) followed by the generation of mutation composite scores (MCS) for each treatment. We also found that MCS is highly treatment specific that MCS derived from one treatment group failed to predict the response in others. Receiver operating characteristics (ROC) analyses showed a superior predictive power of MCS compared to TMB and PD-L1 status for immune therapy-treated patients. Mutation interaction analysis also identified novel co-occurring and mutually exclusive mutations in each treatment group. Our work highlights how patients’ sequencing data facilitates the clinical selection of optimized treatment strategies.

## Introduction

Lung cancer is the leading cause of cancer-related deaths in both men and women in the United States, with an estimated 130,000 deaths in 2022^1^. Non-small cell lung cancer (NSCLC) accounts for 85% of lung cancer cases, which can be further categorized based on their pathological features as adenocarcinoma, squamous cell carcinoma, and large cell undifferentiated carcinoma^2^. Chemotherapy (chemo), Immune checkpoint inhibitors (ICI), and combination therapy of chemo+ICI (chemo+ICI) are the three main treatments used in the clinic for high-stage NSCLC patients based on their health condition, presence or absence of druggable mutation, and expression of immune-related genes^3,4^. Recently, the standard of care for advanced NSCLC without targetable mutations has shifted from chemo to ICI-based regimens, including treatments using single ICI, dual ICI, and chemo+ICI^5^. The chemo+ICI combination treatment has been shown to inhibit tumor growth synergistically with a reduced side effect compared to chemo alone^6^. A combination of Pembrolizumab with pemetrexed and platinum chemotherapy was approved as a first-line treatment for metastatic NSCLC in 2018. Four years later, FDA also approved nivolumab with platinum-doublet chemotherapy for treating patients with resectable NSCLC.

Detection of somatic mutations or other genetic alterations by sequencing has become a standard test that guides clinicians in choosing the right treatments for patients, known as precision medicine^7^. However, whole exome sequencing is time-consuming and not yet cost-effective, restricting its use in the clinic^8^. Therefore, targeted sequencing has been performed as a surrogate approach to identify potential oncogenic drivers and druggable targets^9^. Foundation Medicine (FM) and Guardant Health (GH) are two major sequencing platforms used to examine the genetic alterations in the Atrium Health Wake Forest Baptist (AHWFB) since 2014. DNA isolated from either tumor biopsy or circulating tumor cells is subjected to exome sequencing for selected genes that are known to be associated with the tumor progression or can be targeted by specific treatments^10^. A study in pancreatic cancer found increased CD8 + T cell infiltration in tumors with mismatch repair (MMR) or homologous recombination (HR) mutations which facilitate antitumor immune activation^11^. In the case of lung cancer, *STK11* and *SMACA4* alterations have identified as two biomarkers that predict the response to PD-1 inhibitors in *KRAS*-mutant lung adenocarcinoma^12,13^. However, whether those mutations can predict the responses of patients with wild-type *KRAS* and whether they are still predictive for patients receiving chemo+ICI remains elusive.

Tumor mutational burden (TMB) calculated from the sequencing results is one of the most common biomarkers to predict the patient’s response to immunotherapy^14^. High TMB has been correlated with a better ICI response due to increased neo-antigen expression on the tumor surface, which can be recognized and eliminated by cytotoxic T cells^15^. Researchers found that mutations in beta-2-microglobulin (B2M) and Janus kinase1/2 (JAK1/2) genes are associated with a decreased response to pembrolizumab treatment by suppressing antigen presentation and interferon signaling in melanoma patients regardless of the high TMB, suggesting that a more specific mutational signature is needed to predict patients’ response to ICI in addition to the TMB^16^. Wu et al. found that age difference is a critical parameter that should be considered when using TMB to predict patients’ response to ICI and that high TMB could predict better durable clinical benefit (DCB) in young NSCLC patients but not in the older group^17^. Moreover, the predictive power of TMB has not been tested in any patients who received chemo+ICI treatments. Therefore, more precise biomarkers are needed to predict the patient’s responses to a specific type of ICI treatment.

In this study, we identified treatment-specific genetic alterations from NSCLC patients who received chemo, ICI, and chemo+ICI using Cox proportional hazard regression models followed by generating an MCS including all detrimental or beneficial mutations from two sequencing platforms. Our study suggests that consideration of genetic alterations before applying specific treatments may improve the clinical outcomes of NSCLC patients.

## Results

Clinicopathological features of the patients

**Fig. 1:**
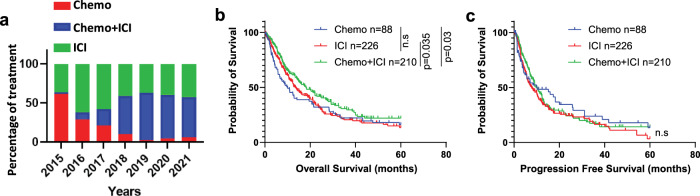
Overview and survival analyses between three treatment groups. **a** Percentage of stage III and IV NSCLC patients who received indicated therapies from 2015 to 2021. **b** Overall survival of patients treated with indicated treatments. **c** Progression-free survival of patients treated with indicated treatments.

We have identified a total of 524 stage III & stage IV NSCLC samples collected at Atrium Wake Forest Baptist Hospital (AWFBH) that either Foundation Medicine sequenced (FM) (*n* = 228) or Guardant Health (GH) (*n* = 296) between 2015 and 2021. Among those 524 patients, 88 were treated with chemotherapy, 226 were treated with ICI, and 210 were treated with chemo+ICI combination therapy. We observed a clear treatment regimen shift from chemotherapy to ICI-based therapy over the last seven years (Fig. 1a). Patients’ characteristics are summarized in Table 1. The mean age was 67.8 years, and 85.1% of patients were white. Most patients had a history of tobacco use (88.9%). Kaplan-Meier analyses showed that patients treated with chemo+ICI had the most prolonged OS (13.1 months) compared to the other two treatment groups (8.4 months for chemo and 11.7 for ICI) (Fig. 1b). Regardless of an extended OS seen in the chemo+ICI group, there was no difference in PFS between those three treatment groups, suggesting that chemo+ICI treated patients had a long-term survival advantage even after tumor stopping respond to the therapy (Fig. 1c).

**Table. 1:** Characteristics of NSCLC patients treated with indicated therapies.

	Foundation Medicine (FM)			Guardant Health (GH)			Total Patients		
	Chemo (*n* = 43)	ICI (*n* = 103)	Chemo+ICI (*n* = 82)	Chemo (*n* = 45)	ICI (*n* = 123)	Chemo+ICI (*n* = 128)	Chemo (*n* = 88)	ICI (*n* = 226)	Chemo+ICI (*n* = 210)
**Sex**									
Female	22 (51.2%)	49 (47.6%)	37 (45.1%)	20 (44.4%)	54 (43.9%)	62 (48.4%)	42 (47.7%)	103 (45.6%)	99 (47.1%)
Male	21 (48.8%)	54 (52.4%)	45 (45.9%)	25 (55.6%)	69 (56.1%)	66 (51.6%)	46 (52.3%)	123 (54.4%)	111 (52.9%)
**Age**									
Mean (SD)	68.7 (9.46)	67.2 (9.12)	66.8 (9.50)	69.7 (10.4)	68.9 (8.86)	67.0 (9.59)	69.2 (9.93)	68.1 (9.04)	66.9 (9.54)
**Race**									
Black	6 (14.0%)	11 (10.7%)	14 (17.1%)	4 (8.9%)	17 (13.8%)	21 (16.4%)	10 (11.4%)	28 (12.4%)	35 (16.7%)
Other	1 (2.3%)	3 (2.9%)	1 (1.2%)	0 (0%)	0 (0%)	0 (0%)	1 (1.1%)	3 (1.3%)	1 (0.5%)
White	36 (83.7%)	89 (86.4%)	67 (81.7%)	41 (91.1%)	106 (86.2%)	107 (83.6%)	77 (87.5%)	195 (86.3%)	174 (82.9%)
**BMI**									
Mean (SD)	25.8 (6.30)	24.3 (5.46)	24.5 (5.22)	25.8 (5.16)	25.3 (7.23)	24.8 (5.20)	25.8 (5.71)	24.8 (6.49)	24.7 (5.20)
**Smoking status**									
Non-Smoker	40 (93.0%)	6 (5.8%)	9 (11.0%)	7 (15.6%)	11 (8.9%)	20 (15.6%)	11 (12.5%)	17 (7.5%)	29 (13.8%)
Smoker	39 (90.7%)	97 (94.2%)	73 (89.0%)	38 (84.4%)	112 (91.1%)	108 (84.4%)	77 (87.5%)	209 (92.5%)	181 (86.2%)
**Stage**									
Three	3 (7.0%)	35 (34.0%)	6 (7.3%)	2 (4.4%)	32 (26.0%)	7 (5.5%)	5 (5.7%)	67 (29.6%)	13 (6.2%)
Four	40 (93.0%)	68 (66.0%)	76 (92.7%)	43 (95.6%)	91 (74.0%)	121 (94.5%)	83 (94.3%)	159 (70.4%)	197 (93.8%)
**TMB**									
<10	12 (27.9%)	78 (75.7%)	53 (64.6%)	0 (0%)	0 (0%)	0 (0%)	12 (13.6%)	78 (34.5%)	53 (25.2%)
10>	4 (9.3%)	23 (22.3%)	28 (34.1%)	0 (0%)	0 (0%)	0 (0%)	4 (4.5%)	23 (10.2%)	28 (13.3%)
Unknown	27 (62.8%)	2 (1.9%)	1 (1.2%)	45 (100%)	123 (100%)	128 (100%)	72 (81.8%)	125 (55.3%)	129 (61.4%)
**PD-L1%**									
High (over 50%)	0 (0%)	29 (28.2%)	16 (19.5%)	0 (0%)	28 (22.8%)	24 (18.8%)	0 (0%)	57 (25.2%)	40 (19.0%)
Low (under 50%)	0 (0%)	34 (33.0%)	56 (68.3%)	1 (2.2%)	34 (27.6%)	57 (44.5%)	1 (1.1%)	68 (30.1%)	113 (53.8%)
Unknown	43 (100%)	40 (38.8%)	10 (12.2%)	44 (97.8%)	61 (49.6%)	47 (36.7%)	87 (98.9%)	101 (44.7%)	57 (27.1%)

Association between mutations and responses in all treatment groups

**Fig. 2:**
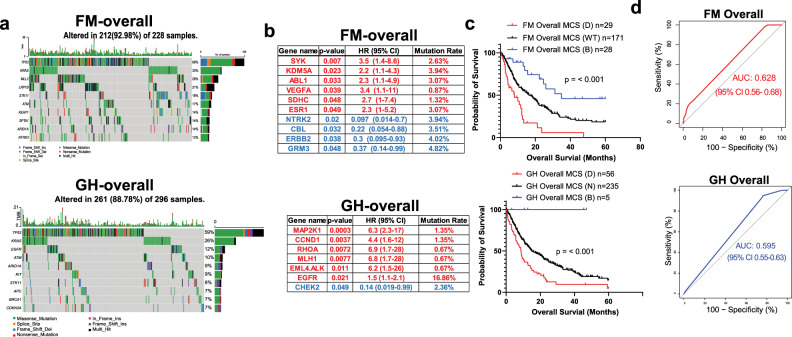
Mutational and survival analyses in all NSCLC patients. **a** Top 10 most mutated genes of overall patients in FM (upper) and GH (lower) cohort. **b** List of mutations that are associated with a worse OS in FM (upper) and GH (lower) cohort. **c** OS of patients with or without detrimental mutations in FM (upper) and GH (lower) cohort. **d** 2-year ROC of FM (upper) and GH(lower) cohort calculated based on MCS.

We first performed multivariant Cox regression analysis, including age, sex, race, BMI, and smoking history in all the patients. We found that high BMI is associated with a worse OS (Supplementary Fig. 1a). Patients were then analyzed separately based on different sequencing platforms, and the top 10 mutated genes in patients who received at least one dose or cycle of the treatment are listed (Fig. 2a). To identify the mutations associated with patients’ OS, we performed univariate Cox proportional hazards analyses by comparing OS between patients with a specific mutated gene and wild-type patients. Among FM-sequenced samples, we identified six detrimental mutations (*SYK, KDM5A, ABL1, VEGFA, SDHC*, and *ESR1*) that were associated with a worse OS (HR > 1, *p* < 0.05) and four beneficial mutations (*NTRK2, CBL, ERBB2*, and *GRM3*) that were correlated with a better OS (HR < 1, *p* < 0.05) (Fig. 2b, upper). We identified six detrimental mutations (*MAP2K1, CCND1, RHOA, MLH1, EML4.ALK, EGFR*) and one beneficial mutation (*CHEK2*) in GH-sequenced patients (Fig. 2b, lower). The position of individual mutation was mapped by lollipop plots (Supplementary Fig. 1b). We then calculated a mutational composite score (MCS) for each patient based on whether they had a detrimental, beneficial, or neither mutation. Among FM-sequenced patients, the median OS in patients carrying at least one beneficial mutation is 34.9 months compared to 7.73 months and 16.36 months in patients with detrimental mutations and wild-type, respectively (Fig. 2c, upper). For those sequenced by GH, patients with at least one beneficial mutation had a median OS of 29 months compared to 8.73 months and 14.96 months in patients with detrimental mutations and wild-type, respectively (Fig. 2c, lower). To test the prediction power of MCS, we plotted the 2-year receiver operating characteristic (ROC) curves based on patients’ MCS. The area under the curve (AUC) is 0.62 for MCS derived from FM and 0.59 for MCS derived from GH (Fig. 2d). Next, we performed somatic mutation interaction analyses for mutations detected by FM and the top 10 most mutated genes. We found that *VEGFA* mutation co-occurred with *MLL2* and *ARID1A* mutations. While mutant *GRM3* co-occurred with mutant *CBL*, *NTRK2, and TP53*. (Supplementary Fig. 1c) We performed the same analysis on the GH-sequenced patients and found three co-occurred mutations: *RHOA* with *CCND1* and *MLH1, CCND1* co-occurred with *CDKN2A*, and *CHEK2* co-occurred with *ATM* (Supplementary Fig. 1d). In addition to mutation, FM and GH provide copy-number alterations (CNAs) data, including deletion and amplification. We only identified three patients in the FM cohort and six in the GH cohort with gene deletions. Therefore, we only focused on testing whether amplification can serve as another prognostic marker. We used the same approach and generated the amplification composite score (ACS) from the FM cohort. We found that the amplification of *CCNE1* and *ZNF217* is correlated with a worse OS (Supplementary Fig. 1e). However, the AUC calculated from the ACS is 0.52, which failed to predict the patients’ response (Supplementary Fig. 1f).

Association between mutations and responses in chemo-treated patients

**Fig. 3:**
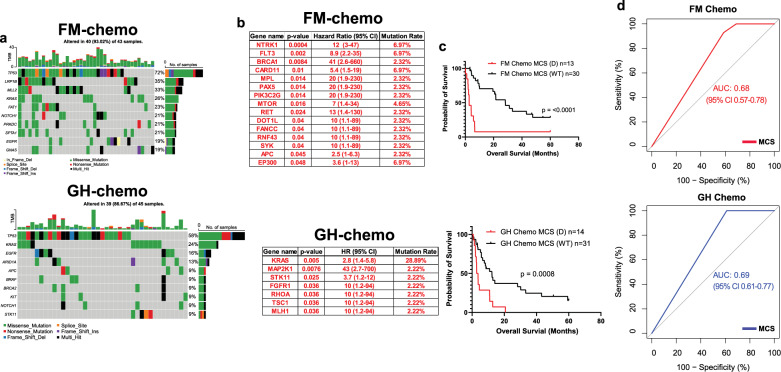
Mutational and survival analyses in chemotherapy treated NSCLC patients. **a** mutation landscapes of chemotherapy treated patients in FM (upper) and GH (lower) cohort. **b** List of mutations that are associated with a worse OS in FM (upper) and GH (lower) cohort of chemotherapy treated patients. **c** OS of patients with or without detrimental mutations in FM (upper) and GH (lower) cohort of chemotherapy treated patients. **d** 2-year ROC of FM (upper) and GH(lower) cohort of chemotherapy treated patients calculated based on their MCS.

Eighty-eight NSCLC patients received chemotherapy, and their treatments were summarized (Supplementary Fig. 2a). Most patients received pemetrexed or carboplatin-based combination chemotherapy. Multivariant Cox regression analysis showed that elderly and male patients had worse OS than younger and female patients (Supplementary Fig. 2b). Patients who received at least one dosage of chemotherapy were then separated based on different sequencing platforms. The top 10 most mutated genes in each sequencing cohort were listed (Fig. 3a). We identified fifteen mutations in the FM cohort and seven in the GH cohort that was significantly correlated with a worse OS (Fig. 3b). Among those 22 detrimental mutations, only five genes (*NTRK1*, *FLT3*, *CARD11*, *EP300*, and *KRAS*) have a mutation rate over 5% and they were mapped by lollipop plotsNotably, 11 out of 13 *KRAS* mutant patients carried G12 single-nucleotide substitutions (9% of G12A, 27% of G12C, 18% of G12D,9% of G12R, and 36% of G12V) (Supplementary Fig. 2c). Patients with at least one detrimental mutation had a worse OS than those who did not carry any of the mutations, and the MCS failed to predict the OS in patients who did not receive chemotherapy (Fig. 3c, Supplementary Fig. 2d). Due to the low mutation rate (<5%) of the genes and limited size of the cohort, most of the co-occurrence mutations were identified in the same patient in the FM cohort (Supplementary Fig. 2e). We identified the co-occurrence of *KRAS* and *STK11* mutations in the GH cohort and both of them were known to be associated with a worse response to chemotherapy (Supplementary Fig. 2f**)**^18,19^. We analyzed the 2-year ROC calculated from chemo MCS and found comparable AUC values between FM (AUC = 0.68) and GH cohort (AUC = 0.69) (Fig. 3d). Lastly, we identified three detrimental amplifications (*AKT2*, *CEBPA*, and *KDR*) associated with a worse OS in the FM cohort (Supplementary Fig. 2g). Again, the AUC calculated from those three amplifications is 0.52, which is insufficient to predict the patients’ response (Supplementary Fig. 2h).

Association between mutations and responses in ICI-treated patients.

**Fig. 4:**
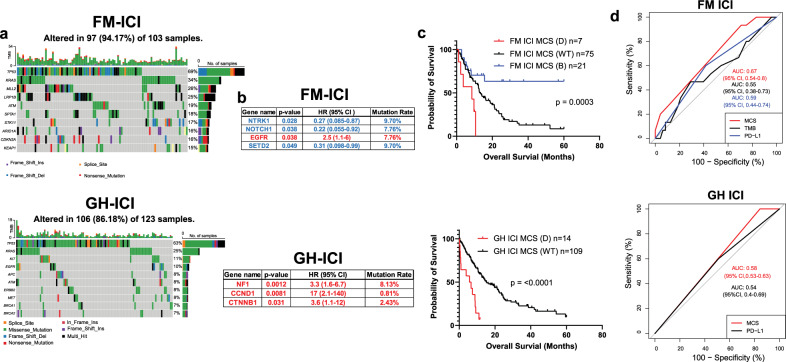
Mutational and survival analyses in ICI treated NSCLC patients. **a** Top 10 most mutated genes of ICI treated patients in FM (upper) and GH (lower) cohort. **b** List of mutations that are associated with a worse OS in FM (upper) and GH (lower) cohort of ICI treated patients. **c** OS of patients with or without detrimental mutations in FM (upper) and GH (lower) cohort of ICI treated patients. **d** Two-year ROC of FM (upper) and GH(lower) cohort of ICI treated patients calculated based on their MCS and TMB.

226 NSCLC patients received one of the five ICI, including pembrolizumab, nivolumab, durvalumab, ipilimumab, and atezolizumab (Supplementary Fig. 3a). Multivariant Cox regression analysis showed that elderly and never smoker patients had a reduced OS (Supplementary Fig. 3b). The top 10 most mutated genes in each cohort were listed in Fig. 4a. We identified three beneficial mutations (*NTRK1*, *NOTCH1*, and *SETD2*) and one detrimental mutation (*EGFR*) that are significantly correlated with the OS in the FM cohort (Fig. 4b, upper). Three harmful mutations (*NF1*, *CCND1*, and *CTNNB1*) were identified in the GH cohort (Fig. 4b, lower). Lollipop plots of individual mutations were mapped on gene structure (Supplementary Fig. 3c). No significant difference of TMB was found in patients with either detrimental or beneficial mutations (Supplementary Fig. 3d). To validate our findings, we used two publicly available ICI datasets sequenced by the MSK-IMPACT panel, Vanguri cohort, and Samstein cohort^20,21^. *EGFR* mutation was validated as a poor prognostic marker in the Vanguri cohort (*n* = 246), and the beneficial effect of *NOTCH1* mutation was validated in the Samstein cohort (*n* = 350) (Supplementary Fig. 3e, f). Although those two cohorts did not validate the beneficial role of SETD2 mutation, mutated *SETD2* was reported to be associated with favorable clinical outcomes in ICI-treated patients^22^. On the contrary, *NF1* mutation was considered a favorable marker for ICI-treated melanoma patients. This contradicts our finding in NSCLC patients, suggesting that the same mutation may play a distinct role in different tumor types^23^. The median OS of patients with *EGFR* mutations was 8.56 months, and the median OS for those with beneficial mutation or wild-type was 25.3 months and 15.26 months, respectively (Fig. 4c, upper). In the GH cohort, the median OS of patients with at least one detrimental mutation is 6.15 months compared to 15.27 months in wild-type patients (Fig. 4c, lower). Again, the ICI-specific MCS failed to predict the OS in patients who did not receive ICI treatment (Supplementary Fig. 3g). Somatic interaction analysis demonstrated that *NOTCH1* mutations significantly co-occurred with *SETD2* mutations, while *EGFR* and *KRAS* mutations were mutually exclusive (Supplementary Fig. 3h). No significant interaction was found in the GH cohort (Supplementary Fig. 3i). Next, we plotted the 2-year ROC in each cohort and found that MCS derived from the FM cohort has a better AUC value (AUC = 0.67) than MCS derived from the GH cohort (AUC = 0.58). We also compared our MCS with established biomarkers including TMB (<10muts/Mb Vs >10muts/Mb) and PD-L1(<50% Vs >50%) and found that MCS has a better prediction value compared to TMB and PD-L1 status in both cohorts (Fig. 4d). Those results suggest that the four mutated genes identified in the FM cohort can be used as a potential surrogate biomarker of TMB to predict the response in ICI-treated patients. Lastly, we identified ten amplified genes associated with a worse OS (Supplementary Fig. 3j). However, the AUC calculated from the ACS (AUC = 0.56) is significantly lower compared to the AUC calculated from the TMB (AUC = 0.66) (Supplementary Fig. 3k).

Association between mutations and responses in chemo + ICI treated patients

**Fig. 5:**
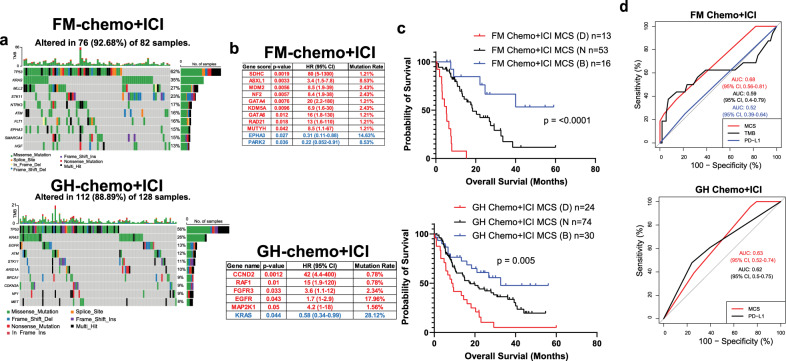
Mutational and survival analyses in chemo + ICI treated NSCLC patients. **a** Mutation landscapes of chemo+ICI treated patients in FM (upper) and GH (lower) cohort. **b** List of mutations that are associated with a worse OS in FM (upper) and GH (lower) cohort of chemo+ICI treated patients. **c** OS of patients with or without detrimental mutations in FM (upper) and GH (lower) cohort of chemo+ICI treated patients. **d** Two-year ROC of FM (upper) and GH(lower) cohort of chemo+ICI treated patients calculated based on their MCS and TMB.

Chemo+ICI combination treatment has been shown to prolong patients’ OS compared to chemo alone group significantly^24^. Two hundred ten patients were treated with chemo+ICI. The majority of them received Carboplatin/Pemetrexed/Pembrolizumab treatment (Supplementary Fig. 4a). Multivariate analysis showed that white patients and patients with a lower BMI responded better than black patients and those who were overweighed (Supplementary Fig. 4b). Top 10 most mutated genes in each cohort were listed (Fig. 5a). We identified nine detrimental mutations (*SDHC*, *ASXL1*, *MDM2*, *NF2*, *GATA4*, *KDM5A*, *GATA6*, *RAD21*, and *MUTYH*) and two beneficial mutations (*EPHA3* and *PARK2)* from the FM cohort (Fig. 5b, upper). Five detrimental mutations (*CCND2*, *RAF1*, *FGFR3*, *EGFR*, *MAP2K1*) and one beneficial mutation (*KRAS*) were identified in the GH cohort (Fig. 5b, lower). The positions of significant mutations were demonstrated by the Lollipop plots (Supplementary Fig. 4c). In the FM cohort, the median OS for a patient carrying a detrimental mutation was 5.33 months compared to 18.36 months and 25.6 months for patients with wild type and beneficial mutations respectively (Fig. 5c, upper). In the GH cohort, the median OS for a patient with a detrimental mutation was 8.8 months compared to 17.5 months and 32.6 months for patients with wild-type and beneficial mutations, respectively (Fig.5c, lower). Again, we did not observe any difference in TMB between patients with either detrimental or benefical mutations (Supplementary Fig. 4d). As we expected, the chemo+ICI-specific MCS failed to predict the OS in patients who received other treatments (Supplementary Fig. 4e). However, we did not find any major co-occurred or mutually exclusive mutations in both cohorts (Supplementary Fig. 4f, g). Next, we plotted the 2-year ROC in each cohort and found that the MCS of the FM cohort has a better AUC value (AUC = 0.69) than MCS derived from the GH cohort (AUC = 0.63). (Fig. 5d). TMB and PD-L1 status had an inferior AUC compared to MCS in both cohorts, suggesting that specific mutation signatures may serve as a better prediction method for patients receiving the chemo+ICI treatments. In addition to the mutation, we identified three amplified genes associated with a worse OS, and the 2-year AUC is 0.54 (Supplementary Fig. 4h, i).

## Discussion

Mutational signatures established from patients have greatly enhanced our understanding of cancer etiology and biological processes involved in tumor initiation, progression, and treatment response^25^. Several studies demonstrated that mutational signatures predict patients’ outcomes independent of known clinical and molecular biomarkers^26^. ICI and chemo+ICI are two primary treatments for high-stage NSCLC patients; however, identifying robust biomarkers to predict the patients’ response remains challenging. Compared to previous studies, our dataset is one of the largest single institute cohorts with comprehensive patient characteristics, treatment regimens, and genomic data, which allows researchers and clinicians to compare and investigate meaningful mutations across different treatment groups and sequencing platforms. To the best of our knowledge, our cohort is the first dataset that includes chemo+ICI-treated patients, which may provide valuable insights into how genetic alterations influence patients’ response to this novel treatment regimen. Chemotherapy drugs modulate the tumor immune microenvironment by either inhibiting immunosuppressive cells or increasing antigen presentation through chemotherapy-induced cell death^27^. Therefore, instead of entirely relying on the cytotoxic effect of chemo to eliminate tumor cells, it jump-starts the cancer-immunity cycle by facilitating antigen release and recognition^28^. Indeed, we found that chemo+ICI significantly improved patients’ OS compared to chemo or ICI alone, even though most of the chemo+ICI treated patients only received less than four cycles of chemotherapy due to adverse effects.

We did not find any overlapped mutations among those three treatment groups, and the mutations derived from one treatment group failed to predict the response in another two groups of patients, indicating various signaling pathways affected by those mutations may play a unique role during the response to chemo and immune activation agents. Interestingly, *KRAS* was identified as a detrimental mutation in the chemo group in the GH cohort, whereas a beneficial mutation in the chemo+ICI group. Numerous studies have shown that patients with *KRAS* mutation had a shorter PFS in response to first-line chemotherapy. The allele difference will also affect the response to different chemotherapies^19,29^. Further validation from another chemo+ICI cohort and in vivo models using *KRAS* mutant syngeneic lines is required to confirm the beneficial effect of *KRAS* mutation in chemo+ICI treated patients. We identified *EGFR* mutation as a prognostic marker for ICI patients with worse OS, further validated by one of the MSKCC cohorts and other published studies^30,31^. We did not observe any hot spot of *NOTCH1* mutation, and further mechanistic studies are crucial to examine whether those mutations are functional. In addition to major mutations, multiple minor mutations (<2%) significantly associated with the patient’s survival were identified from each group. Hence, to better predict the response in patients carrying those minor mutations, we developed a novel prediction algorithm using a composite score calculated from a group of genes, including those with rare mutations. This approach enables us to predict the treatment response in the broader population than using individual genes. Unfortunately, we cannot validate those minor mutations in another two cohorts due to a limited number of patients.

Our data showed that the predictive power of TMB is much higher in the ICI group compared to the chemo+ICI group, suggesting that instead of using overall mutation, mutational signatures composed from essential genes may serve as a better predictor for patients undergoes chemo+ICI treatment. On the other hand, our chemo+ICI specific mutations achieved an AUC of 0.71and further incorporation of extra data such as risk factors identified by multivariate analysis, CT scans, and digitized PD-L1 IHC would further improve our current prediction model. Compared to the robust predictive power of mutational signatures, amplification signatures failed to predict the outcome of any treatment groups. Again, due to the low amplification frequency and limited size of our cohort, further validation in a larger dataset is suggested before drawing any conclusions.

Because of the differences in sample sources, size of gene panels, and under or over-represented tumor clones of biopsy tissue, we did not find any overlapped detrimental or beneficial mutations within the same treatment group sequenced by two platforms. Chae et al. reported a concordance rate of 11.8–17.1% between tissue and blood-based NGS, and over 50% of mutations were not detected in either sequencing method^32^. Another group found that tissue-based NGS detects significantly more genetic alterations compared to plasma-based NGS in lung cancer patients, suggesting that sequencing of tissue samples should be prioritized for molecular testing when tissue is available^33^. Indeed, we validated 3 out of 4 mutations in the ICI FM cohort but not in the ICI GH cohort, and mutations identified from the FM cohort have a much higher AUC than those derived from the GH cohort in both ICI and chemo+ICI groups. However, future validation in larger data sets with matching sequencing platforms or sequencing data derived from both tissue and bood of a same patient are required to cross validate the findings.

To summarize, our results demonstrate the potential usage of mutation data to guide the treatment of NSCLC. Since tumor sequencing data has become increasingly integrated into patients’ medical records, we believe that genomic data will play a vital role in selecting the most appropriate and effective treatment.

## Methods

### Data acquisition

The institutional review board approved the present study at Wake Forest School of Medicine (IRB00078147). Patients were retrospectively evaluated for this study if they had a primary Non-Small Cell Lung Cancer (NSCLC) diagnosis and had genomic profiling performed at AWFBH Health between January 2015 to November 2021. All samples were collected from newly diagnosed patients and patients who received more than one treatments were excluded. Sociodemographics, clinical characteristics, and outcomes were determined via electronic medical records. PD-L1 expression was determined by immunohistochemistry (IHC) performed on FFPE or frozen tissue sections. PD-L1 expression was determined as low (1–49%), or high (≥50%) of total positively stained tumor cells. Data of Tumor Mutational Burden (TMB) were provided by Foundation Medicine. The PFS is calculated based on RECIST 1.1. Written informed consent approved by the Wake Forest Cancer Center Institutional Review Board was obtained prior to sample collection.

### Genomic profiling

Genomic profiling was conducted using next-generation sequencing of circulating tumor DNA (Guardant360 CDx, Guardant Health, Redwood City, CA) and tumor tissue (FoundationOne CDx, Foundation Medicine, Cambridge, MA). All molecular testing were performed in CLIA-compliant labs.

### Generation of mutation composite score (MCS)

Genetic signatures were created based on Overall Survival (OS) stratified by treatment types. The approach consisted of a four-step process. The first step was to screen the genes using Cox proportional hazard regression models to identify any genes that had at least a marginally significant association (*p* < 0.05) with the outcome (OS). Next, the genes identified were separated into “detrimental” and “beneficial” categories based on their association with outcome (i.e., if the presence of a gene was associated with shorter survival (HR > 1), it was considered “detrimental,” whereas if the presence of a gene was associated with more prolonged survival (HR < 1), it was considered “beneficial”). For the third step, each detrimental gene received a score of -1, and each beneficial gene received a score of +1. All genes were weighted equally and all patients were included regardless of their driver mutation status.These scores were then summed across genes. Finally, the overall gene score was created by transforming the gene scores into a 3–level ordinal variable as follows: if the MCS for an outcome was negative, it was grouped as detrimental (−1 group); if the MCS was 0, it was considered wild-type or neutral if both beneficial and detrimental mutations are identified (Note: an MCS score of 0 also would occur if none of the beneficial or detrimental genes were present for a patient), and if the MCS for an outcome was positive (+1 group) it was grouped as protective.

### Statistical analysis

Multivariate analyses were calculated by R glmnet package. To perform OS analysis, we applied Cox proportional hazard regression models with the time between the treatment start date and the date of death. For PFS analysis, we calculated the time between the treatment start date and the day of progression. Hazard ratios and corresponding 95% confidence intervals were estimated from these proportional hazard regression models. GraphPad Prism version 8.4 was used to perform all survival analyses. For gene mutation co-occurrence and mutual exclusion Analysis, we examined the co-occurrence of genes in the signature and the top ten mutated genes in each treatment group. Fisher’s exact test was applied to detect significant somatic interactions. The Maftools package analyzed the mutation patterns and visualized the results in R 4.1.3^34^. For the Receiver operator characteristic (ROC) analysis, ROC curves were generated to test the predictive value of 2-year OS by using MCS, TMB, and response. The area under the curve (AUC) was calculated using the R pROC package^35^. For the forest plot analysis, univariate analysis was performed in different clinical subgroups based on the presence or absence of mutation signatures. A *p*-value <0.05 was used in all analyses to determine significance.

### Reporting summary

Further information on research design is available in the Nature Research Reporting Summary linked to this article.

## Supplementary information

Supplementary information

REPORTING SUMMARY

## Data Availability

The data that support the findings of this study are available from the corresponding author upon reasonable request.
