# Supplementary material for: Prognostic Mutational Signatures of NSCLC Patients treated with chemotherapy, immunotherapy and chemoimmunotherapy
Source: NPJ Precis Oncol. 2023 Mar 27;7:34. doi: 10.1038/s41698-023-00373-0 (PMC10042886; doi:10.1038/s41698-023-00373-0)
Supplement: Supplementary file 1 — Supplementary information [file 41698_2023_373_MOESM1_ESM.pdf]

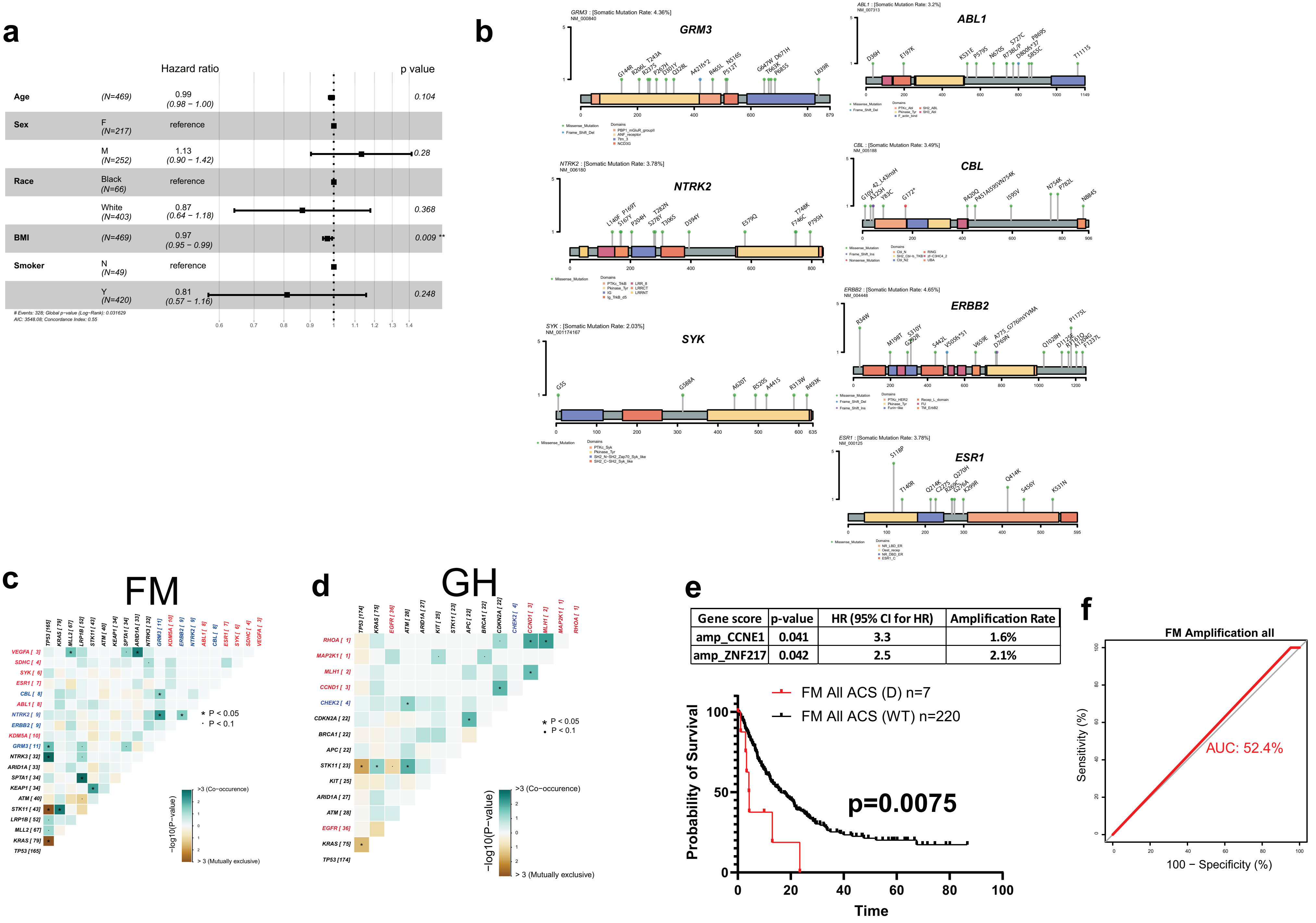

Supplementary Fig. 1 Multivariate ,mutational and ROC analyese of all NSCLC patients

(a) Multivariate analysis of overall patients based on their OS. **(b)** Lollipop plots of major beneficial and detrimental mutations identified from FM and GH cohorts. **(c)** Analysis of co-occurring and mutually exclusive mutations with other major mutations in FM cohort of all patients. **(d)** Co-occurring and mutually exclusive mutations with other major mutations in GH cohort of all patients. **(e)** Amplifications that are associated with a worse OS in FM cohort. **(f)** 2-year ROC calculated from amplifications identified from FM cohort.

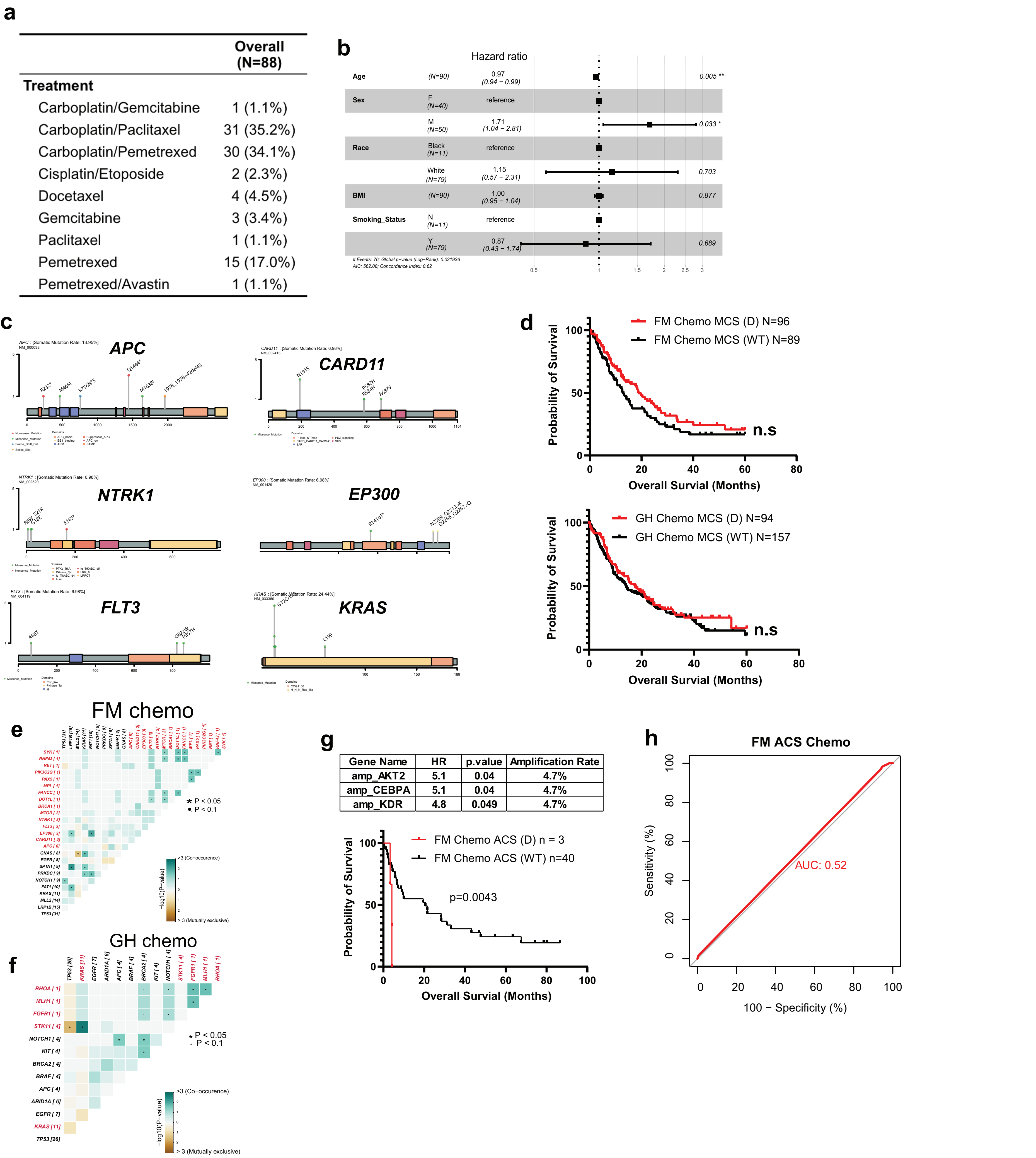

	Overall (N=88)
**Treatment**	
Carboplatin/Gemcitabine	1 (1.1%)
Carboplatin/Paclitaxel	31 (35.2%)
Carboplatin/Pemetrexed	30 (34.1%)
Cisplatin/Etoposide	2 (2.3%)
Docetaxel	4 (4.5%)
Gemcitabine	3 (3.4%)
Paclitaxel	1 (1.1%)
Pemetrexed	15 (17.0%)
Pemetrexed/Avastin	1 (1.1%)

Gene Name	HR	p.value	Amplification Rate
amp_AKT2	5.1	0.04	4.7%
amp_CEBPA	5.1	0.04	4.7%
amp_KDR	4.8	0.049	4.7%

Supplementary Fig. 2 Multivariate ,mutational and ROC analyese of chemotherapy treated NSCLC patients

(a) Treatment summary of patients received different chemotherapy. **(b)** Multivariate analysis of chemotherapy treated patients based on their OS. **(c)** Lollipop plots of major detrimental mutations identified from FM and GH cohorts. **(d)** OS of patients who did not receive chemotherapy with the presence or absence of chemo specific mutations. **(e)** Co-occuring and mutually exclusive mutations with other major mutations in chemo treated patients of FM cohort. **(f)** Co-occuring and mutually exclusive mutations with other major mutations in chemo treated patients of GH cohort. **(g)** Amplifications that are associated with a worse OS in FM cohort. **(h)** 2-year ROC calculated from amplifications identified from FM cohort.

a

	Overall (N=226)
**Treatment**	
Atezolizumab	6 (2.7%)
Durvalumab	41 (18.1%)
Nivolumab	88 (38.9%)
Nivolumab/Ipilimumab	4 (1.8%)
Pembrolizumab	87 (38.5%)

b

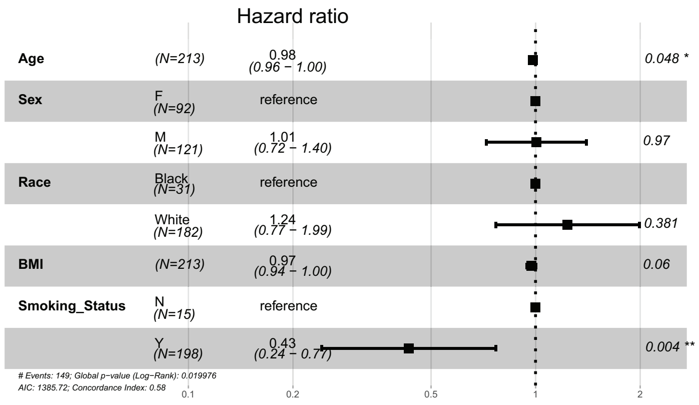

c

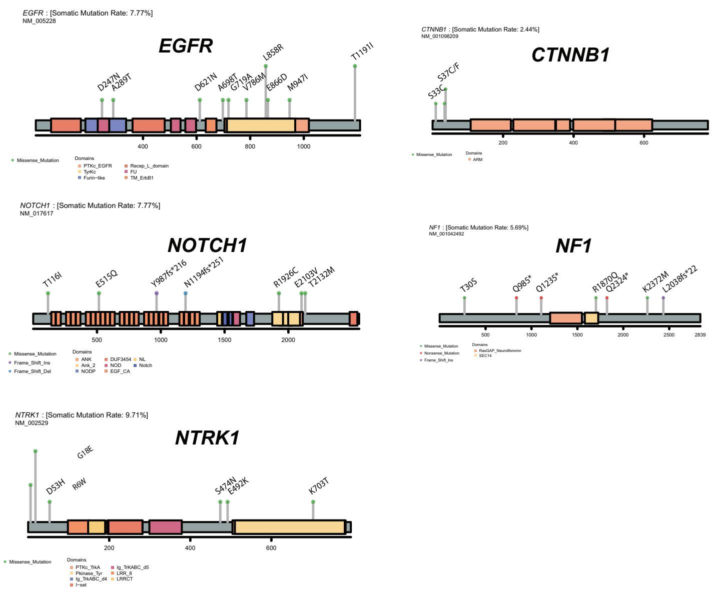

d

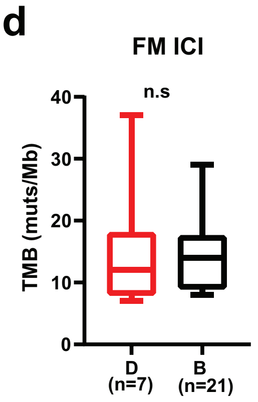

e

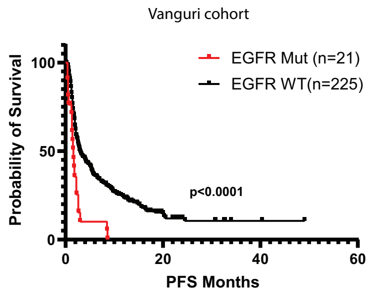

f

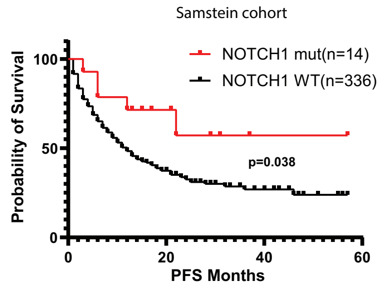

g

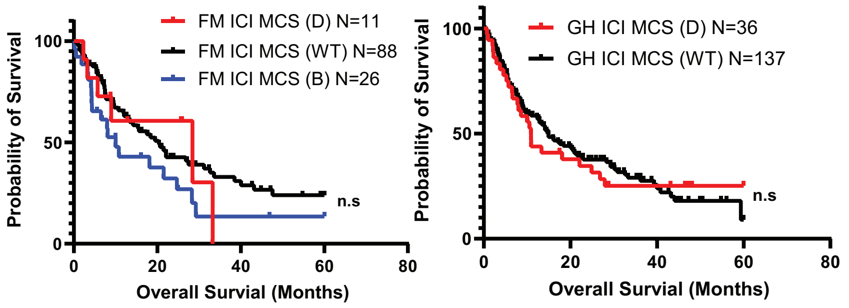

h

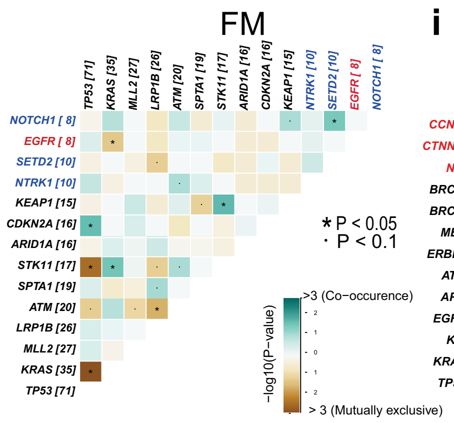

i

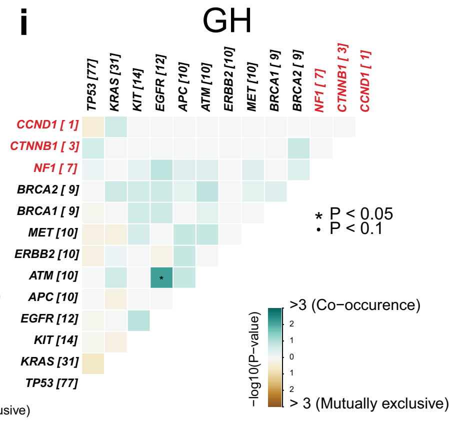

j

Gene Name	HR	p.value	Amplification Rate
amp_KDR	6.3	0.00056	3.9%
amp_ARFRP1	50	0.0014	1.0%
amp_CDK4	7	0.0017	2.9%
amp_KIT	5.3	0.0056	2.9%
amp_PDGFRA	5.3	0.0056	2.9%
amp_BRAF	20	0.0063	1.0%
amp_KEL	20	0.0063	1.0%
amp_SMO	20	0.0063	1.0%
amp_WT1	20	0.0063	1.0%
amp_NFE2L2	3.7	0.029	2.9%

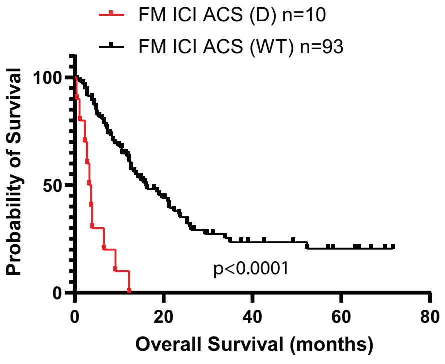

k

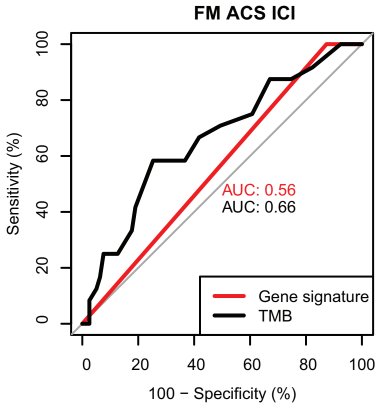

Supplementary Fig.e 3 Multivariate ,mutational and ROC analyese of ICI treated NSCLC patients

(a) Treatment summary of patients received different ICI. **(b)** Multivariate analysis of ICI treated patients based on their OS. **(c)** Lollipop plots of major detrimental mutations identified from FM and GH cohorts. **(d)**TMB of ICI treated patients with either detrimental (D) or beneficial (B) mutations in FM cohort. Center line represents the median value, lower and upper bound of box indicate lower quartile and upper quartile, and whiskers indicate the minimum and maximum values. **(e)**Kaplan-Meier analysis of ICI treated NSCLC patients with or without EGFR mutation using Vanguri cohort. **(f)** Kaplan-Meier analysis of ICI treated NSCLC patients with or without NOTCH1 mutation using Samstein cohort. **(g)** OS of patients who did not receive ICI with the presence or absence of ICI specific mutations. **(h)** Co-occurring and mutually exclusive mutations with other major mutations in ICI treated patients of FM cohort. **(i)** Co-occurring and mutually exclusive mutations with other major mutations in ICI treated patients of GH cohort. **(j)** Amplifications that are associated with a worse OS in FM cohort. **(k)** 2-year ROC calculated from amplifications identified from FM cohort.

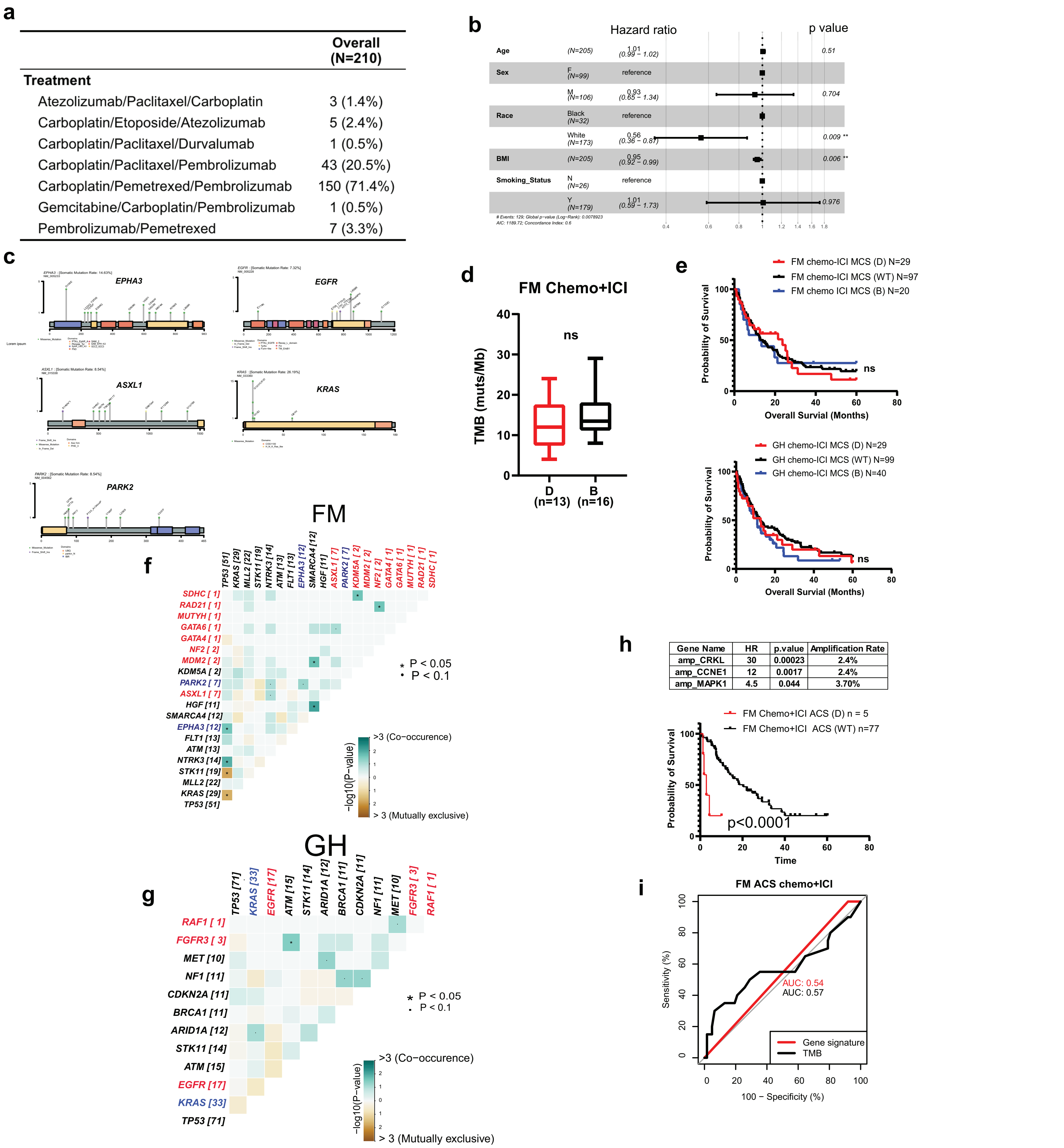

Supplementary Figure 4 Multivariate ,mutational and ROC analyese of chemo+ICI treated NSCLC patients

(a) Treatment summary of patients received different ICI+chemo therapies. **(b)** Multivariate analysis of chemo+ICI treated patients based on their OS. **(c)** Lollipop plots of major detrimental and beneficial mutations identified from FM and GH cohorts. **(d)**TMB of chemo+ICI treated patients with either detrimental (D) or beneficial (B) mutations in FM cohort. Center line represents the median value, lower and upper bound of box indicate lower quartile and upper quartile, and whiskers indicate the minimum and maximum values. **(e)** OS of patients who did not receive chemo+ICI with the presence or absence of chemo+ICI specific mutations. **(f)** Co-occurring and mutually exclusive mutations with other major mutations in ICI treated patients of FM cohort. **(g)** Co-occurring and mutually exclusive mutations with other major mutations in ICI treated patients of GH cohort. **(h)** Amplifications that are associated with a worse OS in FM cohort. **(i)** 2-year ROC calculated from amplifications identified from FM cohort.
